# Multicentric standardization of minimal/measurable residual disease in B‐cell precursor acute lymphoblastic leukaemia using next‐generation flow cytometry in a low/middle‐level income country

**DOI:** 10.1111/bjh.18499

**Published:** 2022-10-12

**Authors:** Maura R. V. Ikoma‐Colturato, Camila M. Bertolucci, Joana E. Conti‐Spilari, Elen Oliveira, Anderson J. Simioni, Lorena L. Figueredo‐Pontes, Felipe M. Furtado, Ana P. Alegretti, Ana P. Azambuja, Fabiola Gevert, Bernadete E. Gomes, Danielle M. V. Avelar, Ana Cláudia C. V. Soares, Priscila M. Ramos, Berta Santos, Maria L. Cortez, Miriam P. Beltrame, Nydia S. Bacal, Andressa Wagner, Norma Lucena‐Silva, Alex F. Sandes, Fernanda Cunha, Gustavo H. M. Oliveira, Elaine S. Costa, Mihoko Yamamoto

**Affiliations:** ^1^ Hospital Amaral Carvalho São Paulo Brazil; ^2^ Sabin Medicina Diagnóstica Brasília Brazil; ^3^ Instituto de Pediatria e Puericultura Martagão Gesteira UFRJ Rio de Janeiro Brazil; ^4^ Division of Hematology, Department of Medical Images, Hematology, and Clinical Oncology, Ribeirão Preto Medical School University of São Paulo Ribeirão Preto Brazil; ^5^ Hospital de Clínicas de Porto Alegre Rio Grande do Sul Brazil; ^6^ Hospital Nossa Senhora das Graças Curitiba Brazil; ^7^ Hospital de Clínicas da Universidade Federal do Paraná Curitiba Brazil; ^8^ Hospital Pequeno Príncipe Curitiba Brazil; ^9^ Instituto Nacional do Cancer Rio de Janeiro Brazil; ^10^ Oncobio, Belo Horizonte Minas Gerais Brazil; ^11^ Diagnósticos da América São Paulo Brazil; ^12^ Santa Casa de Porto Alegre Rio Grande do Sul Brazil; ^13^ Diagnósticos da América Rio de Janeiro RJ Brazil; ^14^ Núcleo de Hematologia Belo Horizonte Minas Gerais Brazil; ^15^ Hospital Infantil Erasto Gaertner Curitiba Brazil; ^16^ Hospital Israelita Albert Einstein São Paulo Brazil; ^17^ Hemocentro de Florianópolis Santa Catarina Brazil; ^18^ Laboratório Santa Luzia Santa Catarina Brazil; ^19^ Instituto de Medicina Integral Professor Fernando Figueira Recife Brazil; ^20^ Grupo Fleury Medicina Diagnóstica São Paulo Brazil; ^21^ Laboratório Sollutio Campinas Brazil; ^22^ Laboratório Getúlio Sales Natal Brazil; ^23^ Universidade Federal de São Paulo (EPM/UNIFESP) São Paulo Brazil

**Keywords:** B cell acute lymphoblastic leukaemia, B‐ALL, flow cytometry, minimal residual disease, MRD, next generation flow

Minimal residual disease (MRD) in acute lymphoblastic leukaemia (ALL) is a strong predictor of prognosis and its identification is incorporated into most ALL treatment protocols. Therefore, MRD assessment methods should have sufficient specificity and sensitivity to detect residual leukaemic cells to be reproducible, enabling multiple centres to use the same strategy effectively.^1^ In Brazil, multiparametric flow cytometry (MFC) is currently the most accessible method for assessing MRD in ALL. However, our previous efforts to harmonize MRD detection in ALL by MFC^2^ found frequent variability in MRD results reported by laboratories. Therefore, we aimed to improve this situation by implementing the B‐cell precursor (BCP)‐ALL MRD Next‐Generation Flow (NGFlow) in our country's laboratories, as it is a fully standardized and interlaboratory validated method.^3^, ^4^ This study was registered under number CAAE45814821.0.1001.5435 at the Ethics Committee.

After a training phase in standard operation procedures (SOPs),^3^, ^5^, ^6^, ^7^ and analysis strategies (Figure S1), the 26 recruited laboratories were requested to submit flow cytometry standard (FCS) files of BCP‐ALL MRD samples for centralized analysis at two brazilian laboratories experienced in EuroFlow standardization (Figure S2). The requirements to participate in the study can be found in the supporting information. In an initial phase of the study, 21 laboratories submitted at least 10 Flow Cytometry Standard (FCS) files per laboratory (*n* = 195) for centralized analyses. In the sequential phase, 16 of the 21 labs submitted an additional 3–5 FCS files each (*n* = 77; Figure S2). The parameters by which the files were compared to the reference values were: MRD results, detection of residual B cells, median fluorescence intensities (MFI) of each NGFlow panel marker for BCP‐ALL MRD expressed in residual B‐cell subsets (Table S1) and medians of forward (FSC) and side (SSC) light scatter of lymphocytes, obtained from FCS files and recorded by Infinicyt software (Cytognos™). The MFI comparability matrices of the panel markers were constructed using 45 BCP‐ALL MRD samples using BD FACSCanto II™ and BDFACSLyric™ (Figure S3). MFI values within the reference mean ± 2 standard deviations (2SD) were considered concordant (Figure S4).

Statistical analyses were performed using the R v4.1.2 software with a significant *p* < 0.05. Pearson's correlation was used for concordance analyses of the MFI results between the two central analysts. MRD agreement was defined as results within one log compared to centralized analyses. The chi‐squared test was used to compare the analysis of MRD and B‐cell subsets between the two phases of the study.

We observed a high correlation between the results of the two central analysts in all parameters evaluated (*R* > 0.95; *p* < 0.001; Figure S5) showing that reproducibility of the method is possible if the same strategies are used. The use of reference matrices allowed data comparability and evaluation of the intra‐ and interlaboratory reproducibility of the method. This evaluation system allowed monitoring the deviations in SOPs and quality control of the reagents,^8^, ^9^ enabling the identification of potential pitfalls and the recommendation of corrective actions (see supporting information). At least 4 × 10^6^ events were acquired in 247/272 (91%) samples. Hypocellular samples did not reach this number, but this was not persistent in individual laboratories. The average number of acquired events was 8.6 × 10^6^ (0.2 × 10^6^ to 20 × 10^6^).

More than 85% of the concordant MRD results were observed in both phases of the study. Higher levels of MRD (between 0.01% and 0.1% and >0.1%) were less discordant, which was improved in the sequential phase of the study (Table  1 ). In disagreement with the centralized analyses, MRD was identified in 3% of the laboratory files in the initial phase, but not in the sequential phase (Table  1). This was attributed to the experience gained, emphasizing the need for expertise to evaluate MRD. Additionally, in both phases of the study, we noticed differences larger than one log in 4% of the FCS files at all MRD positivity levels, always from the same laboratories, indicating the need for retraining (Table  1; Figure S2). Discordant MRD results were mainly observed at levels below 0.01%, in 6% and 10% of samples in the initial and sequential phases respectively (Table 1). This was not related to specific immunophenotypes, but reflected greater difficulty in detecting small numbers of leukaemic cells. Although current treatments use a MRD cut‐off level of 0.01%, detection of residual leukaemic cells below this level is of prognostic significance and should be monitored.^10^, ^11^ Concordant results between laboratories and central analyses were observed for the detection of normal BCP and mature B cells in both the initial (93%) and sequential (97%) phases of the study (Table 2). Some discrepancies observed were attributed to the difficulties in detecting small cell populations and also to the misinterpretation of regenerative B cells as residual blasts. The agreement of MFI markers in normal B cells with the central analysts was greater than 80% in CD10, CD19, CD34, CD38 and lower in CD20, CD45 and CD81 (76%, 58% and 70% respectively); this was improved in the sequential phase (Table S2; Figure S6). Lymphocytes’ FSC and SSC light scatter were the most discrepant results from the Euroflow SOP’s target values,^3^ indicating inappropriate settings of these parameters. No significant difference was observed between the two phases of the study (Table S2; Figure S6).

**TABLE 1:** Comparison of MRD results in B‐cell acute lymphoblastic leukaemia patients between centralized and participating laboratories evaluated in the initial and sequential phases of the study

MRD detection	Initial phase^a^ (*N* = 195)	Sequential phase^a^ (*N* = 77)	*p* value^b^
Centralized analyses vs participating laboratories	*n* (%)	*n* (%)	
Total of concordant results^c^	168 (86)	66 (86)	
Total of discordant results	27 (14)	11 (14)	
MRD^neg^ vs MRD^pos^	6 (3)	0	
MRD^pos^ vs MRD^neg^	12 (6)	8 (10)	NS
MRD <0.01%	8 (67)	7 (87)	
0.01 < MRD < 0.1%	3 (25)	1 (12)	
MRD >0.1%	1 (8)	0	
MRD^pos^ vs MRD^pos^ (difference >1 log)	9 (4)	3 (4)	

Abbreviation: FCS, Flow Cytometry Standard; MRD, minimal residual disease; neg, negative; pos, positive.

^a^ Initial phase: 19 laboratories [195 FCS files]; sequential phase: 16 laboratories (77 files).

^b^ Pearson's chi‐squared test.

^c^ Concordance: within one log of the reference MRD percentage.

**TABLE 2:** Comparison of data analysis results of the residual normal B cells in MRD samples from B‐cell acute lymphoblastic leukaemia patients between centralized and participating laboratories

B‐cell subset analysis	Initial phase^a^ (*N* = 195)	Sequential phase^a^ (*N* = 77)	*p* value^c^
Centralized analyses versus participating laboratories	*n* (%)	*n* (%)	
Concordant results			
B‐cell precursors	182 (93)	75 (97)	NS
Mature B cells	188 (96)	75 (97)	
Discordant results^b^			
BCP^pos^ vs BCP^neg^	6 (3)	0	NS
BCP^neg^ vs BCP^pos^	7 (3)	2 (3)	
MBC^pos^ vs MBC^neg^	6 (3)	1 (1)	
MBC^neg^ vs MBC^pos^	1 (1)	1 (1)	

Abbreviations: BCP, B‐cell precursors; FCS, Flow Cytometry Standard; MBC, mature B cells; MRD, minimal residual disease.

^a^ Initial phase: 19 laboratories (195 FCS files); sequential phase: 16 laboratories (77 FCS files); pos: positive, neg: negative.

^b^ Pearson's chi‐squared test.

^c^ Centralized results versus participating laboratories results.

Improvements in longitudinal intra‐laboratory concordant results were also observed in the sequential versus initial phase for most parameters (Figure S7). The performance of some laboratories was variable during the study, reflecting non‐strict adherence to SOPs over time (Figure S7) and indicating the need for continuous quality assessment, complemented by educational programmes on the standardization of methods even for experienced laboratories, as previously demonstrated.^12^, ^13^

Our results showed that standardization resulted in a greater accuracy in the MRD evaluation of BCP‐ALL in different laboratories and on different MFC platforms, as previously described,^14^ and also that MFC reproducibility is possible in a multicentre study if the laboratories adhere to the proposed SOPs. Comparability between samples will also be essential to enable automated analysis using the EuroFlow database.^3^ In conclusion, NGFlow was effective in minimizing the differences in BCP‐ALL MRD results observed in Brazil, with the sensitivity and accuracy necessary for clinical applicability. The strategies used here can be useful for low‐ and middle‐income countries, even those with continental territories.

## AUTHOR CONTRIBUTIONS

Maura R. V. Ikoma‐Colturato designed the study, performed the educational programme, centralized analysis, and drafted the manuscript; Joana E. Conti‐Spilari and Camila M. Bertolucci constructed the matrices of reference values, performed the educational programme and did part of the data analysis; Elen Oliveira performed centralized analysis, Elaine S. Costa assisted in the study design, Anderson J. Simioni performed statistical analysis, Mihoko Yamamoto provided critical revision of the manuscript for important intellectual content. All other authors performed flow cytometry data analysis and reviewed the manuscript.

## CONFLICT OF INTERESTS

The authors declare no competing interests.

## Supporting information


Figure S1

Figure S2

Figure S3

Figure S4

Figure S5

Figure S6

Figure S7

Appendix S1

Table S1

Table S2

## Data Availability

The data that support the findings of this study are openly available in the manuscript and in the supporting information of this article.
