# Supplementary figures and images for: Multicentric standardization of minimal/measurable residual disease in B‐cell precursor acute lymphoblastic leukaemia using next‐generation flow cytometry in a low/middle‐level income country
Source: Br J Haematol. 2022 Oct 12;200(3):381–4. doi: 10.1111/bjh.18499 (PMC10091773; doi:10.1111/bjh.18499)

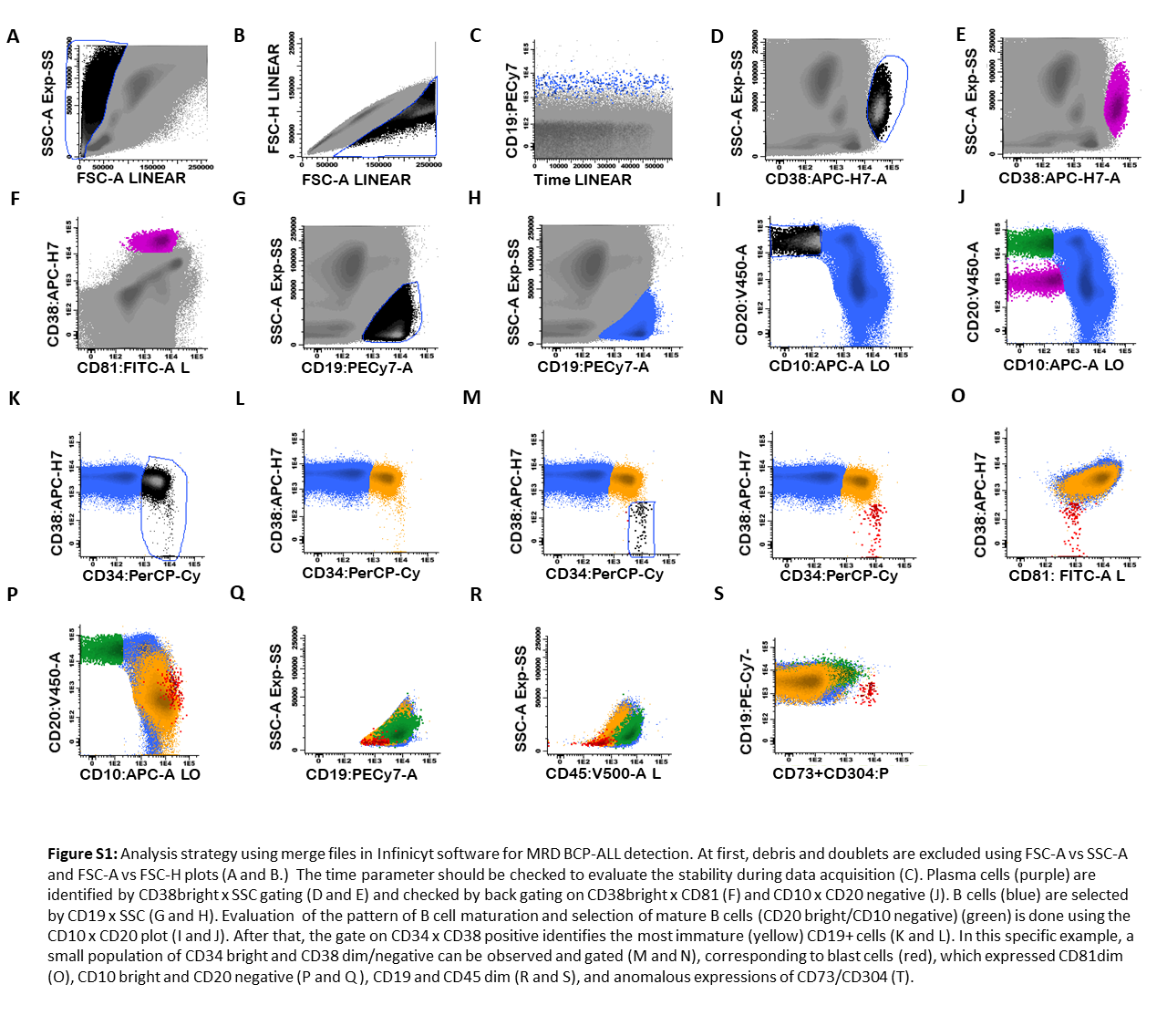

Supplement: Supplementary file 1 — Figure S1 [file BJH-200-381-s005.TIF]

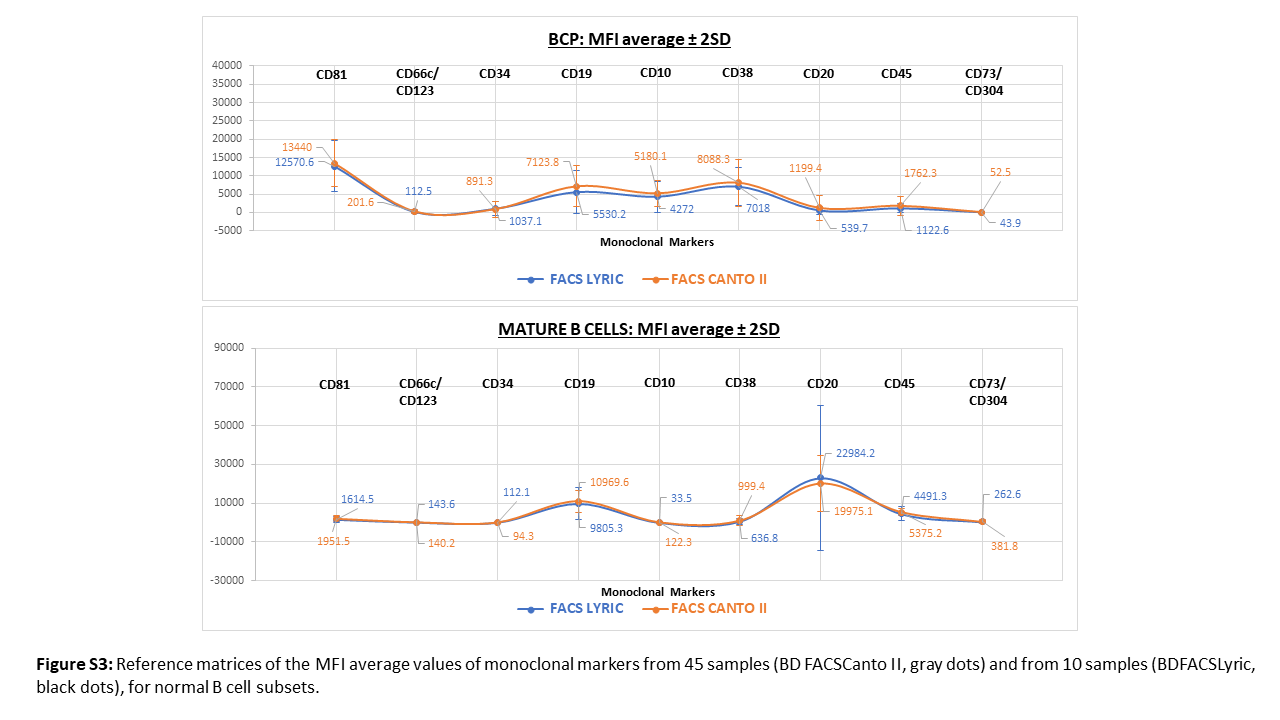

Supplement: Supplementary file 3 — Figure S3 [file BJH-200-381-s009.TIF]

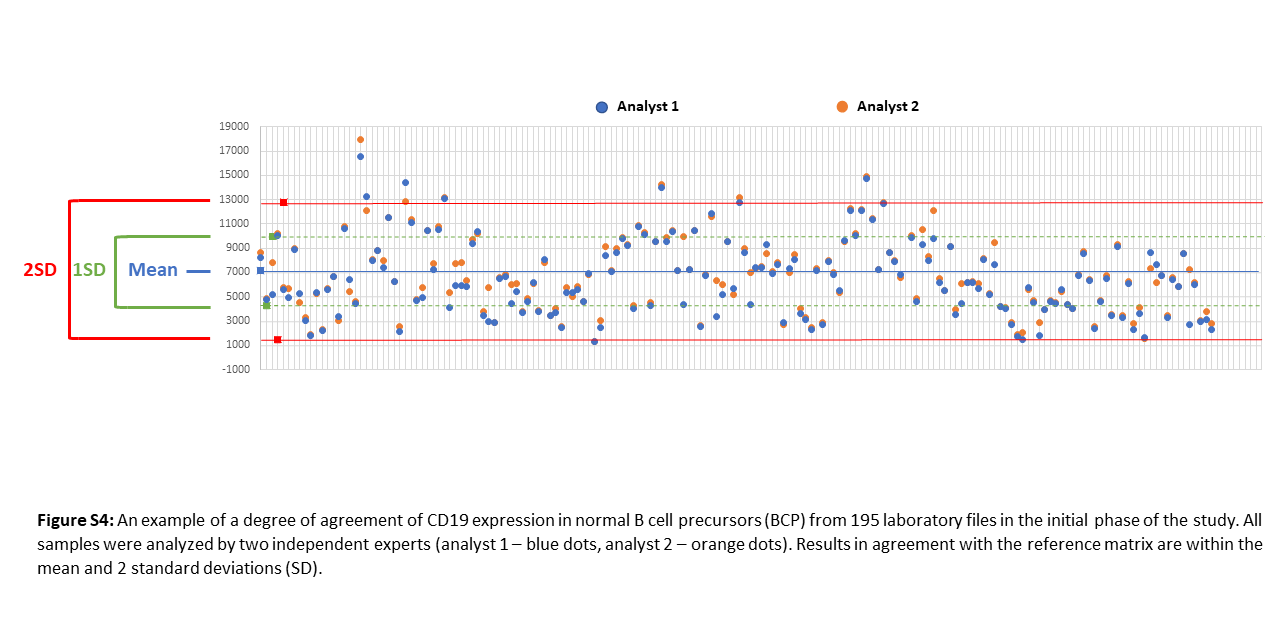

Supplement: Supplementary file 4 — Figure S4 [file BJH-200-381-s002.TIF]

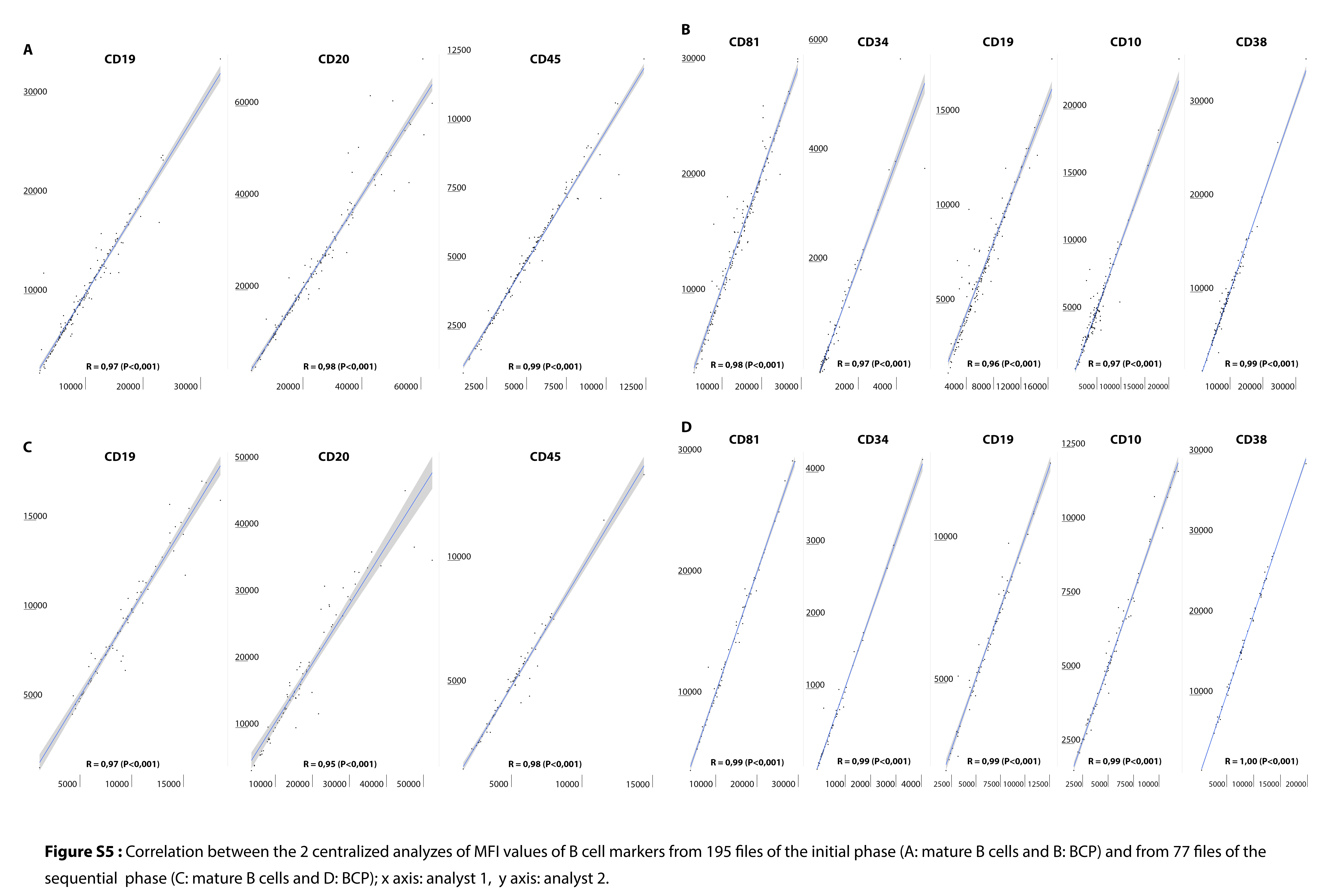

Supplement: Supplementary file 5 — Figure S5 [file BJH-200-381-s003.png]

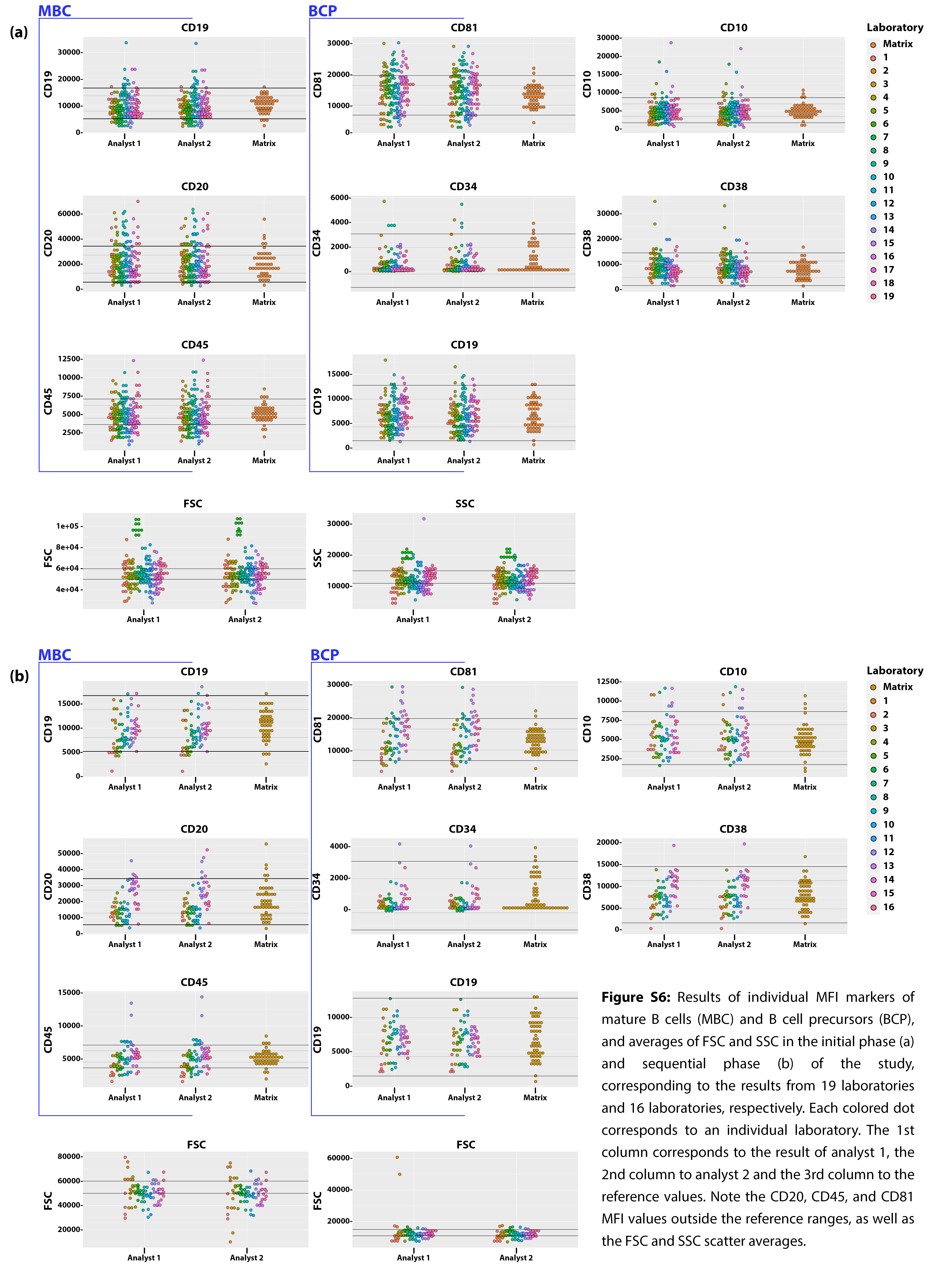

Supplement: Supplementary file 6 — Figure S6 [file BJH-200-381-s004.png]

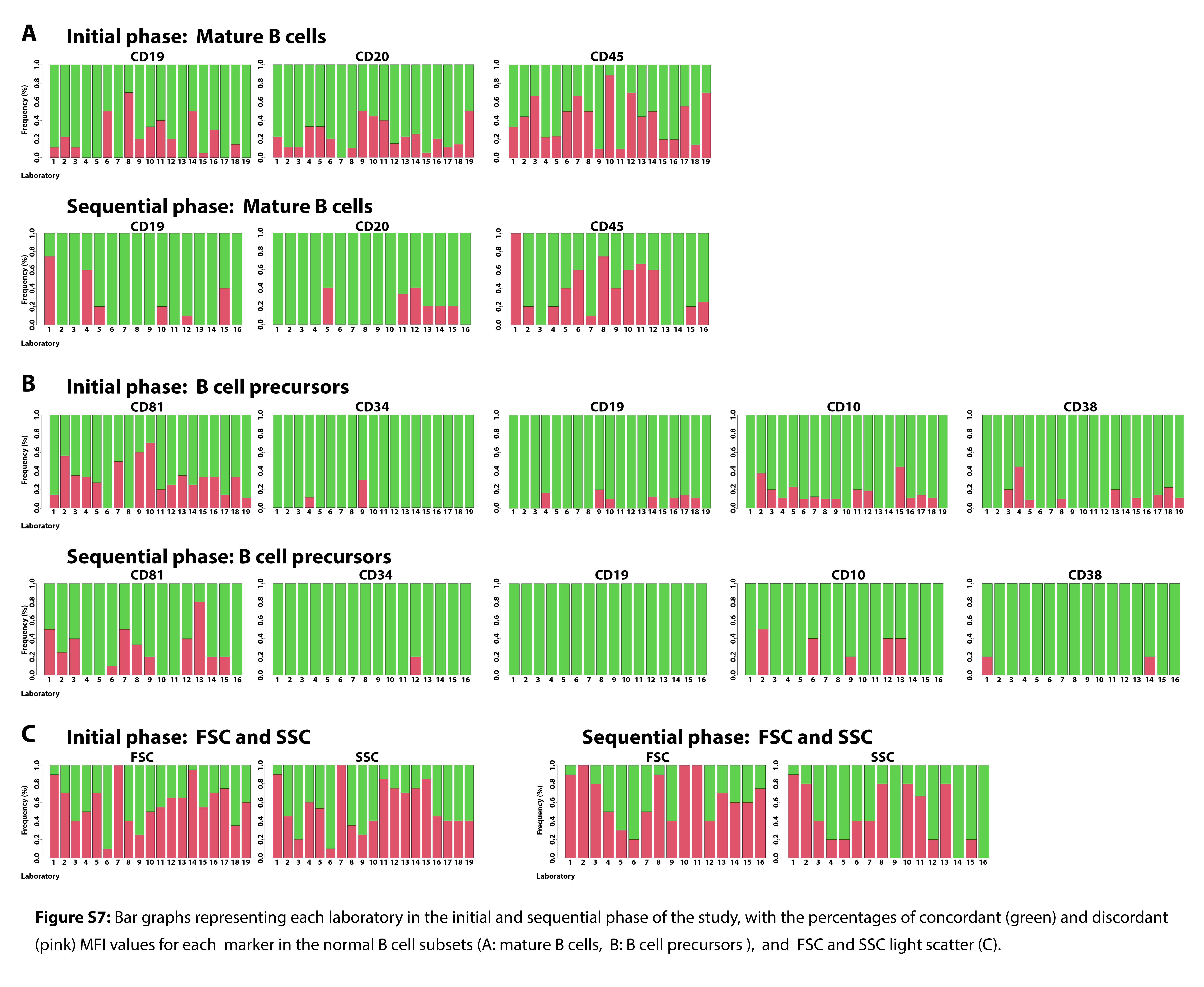

Supplement: Supplementary file 7 — Figure S7 [file BJH-200-381-s006.png]
