# Supplementary material for: Multicentric standardization of minimal/measurable residual disease in B‐cell precursor acute lymphoblastic leukaemia using next‐generation flow cytometry in a low/middle‐level income country
Source: Br J Haematol. 2022 Oct 12;200(3):381–4. doi: 10.1111/bjh.18499 (PMC10091773; doi:10.1111/bjh.18499)
Supplement: Supplementary file 2 — Figure S2 [file BJH-200-381-s007.pptx]

## Slide 1
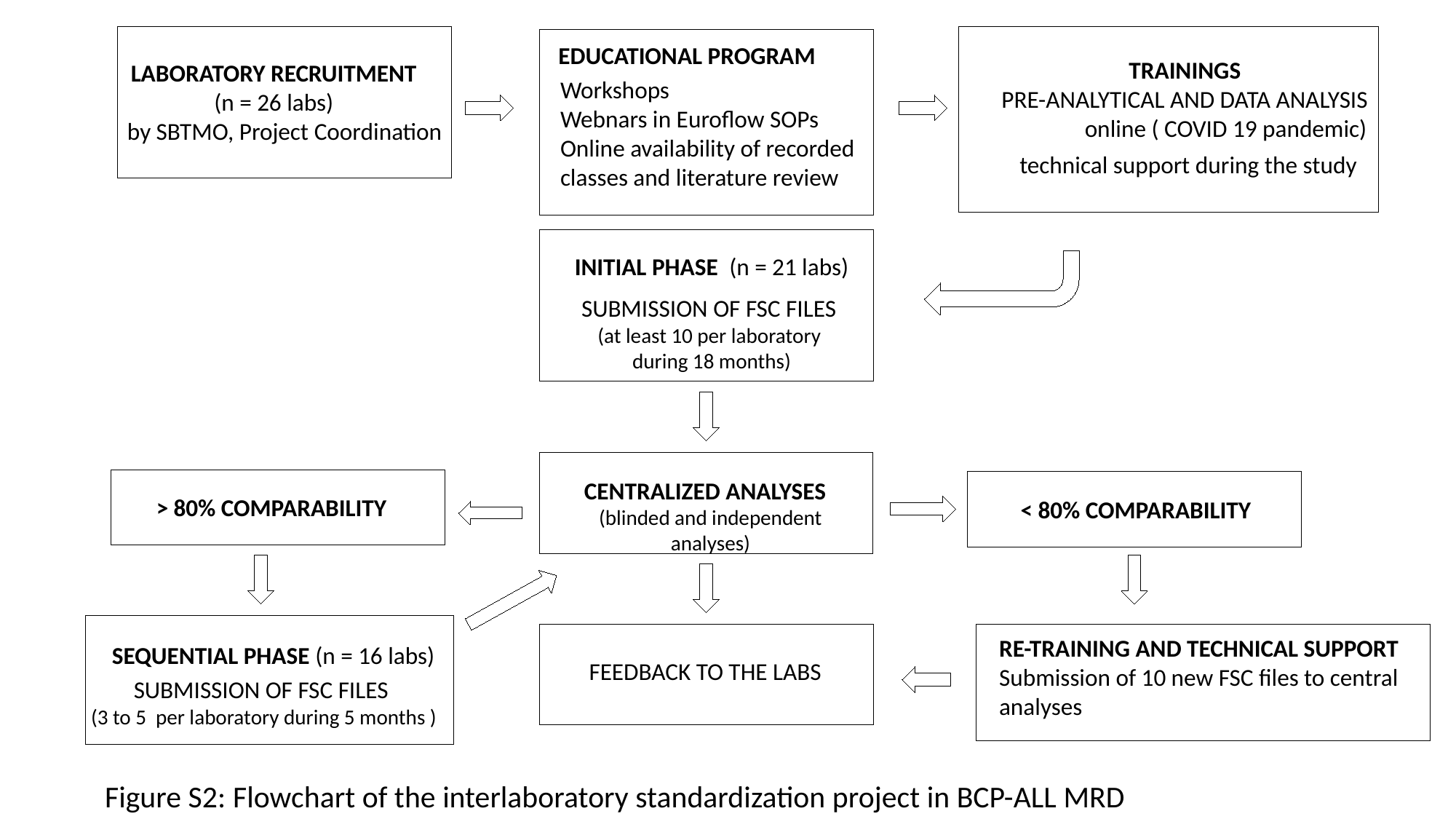

LABORATORY RECRUITMENT
(n = 26 labs)
 by SBTMO, Project Coordination
Trainings
pre-analytical and data analysis
 online ( COVID 19 pandemic)
technical support during the study
EDUCATIONAL PROGRAM
Workshops
Webnars in Euroflow SOPs
Online availability of recorded classes and literature review
INITIAL PHASE (n = 21 labs)
SUBMISSION OF FSC FILES
(at least 10 per laboratory
during 18 months)
CENTRALIZED ANALYSES
(blinded and independent analyses)
> 80% COMPARABILITY
< 80% COMPARABILITY
Feedback to the labs
RE-TRAINING AND TECHNICAL SUPPORT
Submission of 10 new FSC files to central analyses
SEQUENTIAL PHASE (n = 16 labs)
SUBMISSION OF FSC FILES
(3 to 5 per laboratory during 5 months )
Figure S2: Flowchart of the interlaboratory standardization project in BCP-ALL MRD
