# Supplementary material for: Multicentric standardization of minimal/measurable residual disease in B‐cell precursor acute lymphoblastic leukaemia using next‐generation flow cytometry in a low/middle‐level income country
Source: Br J Haematol. 2022 Oct 12;200(3):381–4. doi: 10.1111/bjh.18499 (PMC10091773; doi:10.1111/bjh.18499)
Supplement: Supplementary file 8 — Appendix S1 [file BJH-200-381-s008.docx]

**SUPPLEMENTAL MATERIALS**

**SUPPORTING INFORMATION**

**Requirements for participating in the study and the** **criteria for inclusion and exclusion of samples**

1. Requirements to participate in the study were to use flow cytometer platforms validated by Euroflow (fifteen participating laboratories used BD FACSCanto ^TM^ II, 3 used BDFACSLyric^TM^, and 3 used both platforms).
2. MRD samples must have been evaluated with full Euroflow standard operating procedures (SOPs) without adaptations regarding reagents, monoclonal antibodies (MoAb) combinations, calibration protocols, fluorescence adjustments, compensation, sample preparation, according to the previous description (1-3). **The data files had to meet the criteria of < 5% blast count by morphology and by flow cytometry, to avoid samples with a large number of blasts, which would escape the objective of evaluating the accuracy in MRD detection.**
3. FCS files should contain both normal mature and precursor B cells to enable the evaluation of B cell markers MFIs, and the detection of these residual cells.
4. Data files from patients with previous therapeutic use of monoclonal antibodies (MoAb), especially anti-CD19 therapies were not considered for evaluation for the current study.
5. MRD assessments could be performed at any stage of the treatment and there was no restriction on the patient's age.
6. **FCS data files should preferably have ≥ 4 million events acquired to obtain greater test sensitivity, but this was not an exclusion criterion. Some samples were collected at times when hypocellularity is expected, such as during induction therapy or early post-hematopoietic stem cell transplantation (HSCT) evaluation. Bulklysis was used to concentrate the sample and reach a sufficient number of acquired cells and to reach a sensitivity of 10^-5^ (2).**

**Recommendations for the quality control of samples and indicators used to evaluate MRD samples**

1. **Instructions for sample collection to avoid hemodilution: first pull of bone marrow aspirate; the total volume must not exceed 2 mL.**
2. **Participating laboratories should not include clotted and hemolyzed samples.**
3. **Samples should preferably be processed within 24 hours of collection, transported and stored at controlled temperature (room temperature) until processing.**
4. **The hemodilution of the samples could be evaluated by the increase in the percentage of CD10+ granulocytes as previously described (4), since neutrophils are the main granulocytes in peripheral blood. But this was not a sample exclusion factor.**
5. **Sample stability was evaluated by the visual inspection of dot plots, that enabled detection of sample compromise by light scatter, the evaluation of antibodies staining and linear non-specific antibody binding, and the quality of the sample acquisition, as described by Wood et al (5).**
6. **The number of events in the debris region was evaluated, which reflects the quality of the bulklysis process and also the sample stability.**
7. **Verification that the correct compensation and fluorescence adjustments have been applied.**
8. **The time parameter has been checked for problems during sample acquisition**
9. **Evaluation of the medians of the MFI of each individual marker and of the FSC and SSC light scatter, comparing with the reference values. Samples should have ≥ 80% of agreement on theses parameters.**

**Recommendations for instrument setup and compensation, reagents, sample preparation and acquisition**

Standardized instrument setup of 8 color FACSCanto II cytometer (Becton/Dickinson Biosciences –BD San Jose, CA) and FACSLyric (BDB) was used according to the recommendation of the EuroFlow SOP for instrument setup and compensation (1,3). In Canto II, the median fluorescence intensity MFI of a reference peak of Rainbow beads calibration particles (target value of the 7th peak) was used to set up photomultiplier (PMT) voltages for each detector channel in individual instruments to reach the same MFI across distinct instruments (6). BDFACSLyric uses a collection of attributes to place the positive population in the same position between instruments in the form of assays as one of its components integrated into the Cytometer Setup and Tracking (CS&T) module (6). EuroFlow Lyric assays enabled the standardization of acquisition of 8 color panels without the need to manually configure the PMT voltages on the individual instruments, but by setting specific tube target values (TTV) for each fluorochrome pair (6). The fluorescence compensation matrix was calculated in each acquisition software such as FACSDiva (BD Biosciences) and FACSSuite (BD Biosciences), respectively for Canto II and Lyric, according to a defined set of single stained compensation tubes as previously described (1,3). Daily monitoring of instrument performance was recommended (1).

***Reagents****:* *Bulklysis Solutions*: Both BD FACS Lysing solution and in-house prepared ammonium chloride were used by the laboratories to perform bulklysis (7)*. Rainbow beads*: EuroFlow validated Rainbow Calibration Particles lots (available in [www.euroflow.org](http://www.euroflow.org)), 8 peaks (Spherotech, Lake Forest, IL) were used for fluorescence adjustment in the BD FACSCanto II instrument setup.

***Monoclonal antibody combination****:* The two eight-color tubes designed by Euroflow for BCP ALL MRD were used in the study. Six markers were common in both tubes (CD81/FITC), CD34/PerCPCy5.5, CD10/APC, CD19/PECy7, CD38/APC750 or APCH7/, CD20/PacB or HV450, CD45/PacO or V500 or OC515, and 2 markers combined in the PE fluorescence channel: CD66c/CD123 in the first tube and CD73/CD304 in the second tube (2). Reference antibodies were used, but alternative MoAbs validated by Euroflow and commercially available in the country were also included to give greater access to the participation of laboratories. MoAb specifications and sources are in Table S1.

***Sample preparation and acquisition***: The protocol included the erythrocyte bulklysis procedure to concentrate the sample and achieve the acquisition of sufficient numbers of cells to reach a sensitivity of 10^-5^ as previously described (2). Bulklysis procedures for bone marrow samples were performed as previously described (7), preferably within 24 hours after collection. Samples were prepared using a stain/lyse/wash method as described in the EuroFlow standard operating procedures (SOPs) (1). Data acquisition was done immediately after sample preparation.

**Data Analysis**

**Laboratories were trained in data analysis as described below. We used the strategy of merging the two data files corresponding to the 2 tubes of the BCP-ALL MRD in the Infinicity software (Cytognos)^TM^, which allow the analysis of a greater number of events. Gate strategies are explained in figure S1**

**Pitfalls detected during the evaluation process that enabled suggestions for corrective actions**

Performance evaluation of multiple flow cytometers and inter-laboratory comparability were possible after laboratories had implemented SOPs for instrument setup, including setting fluorescence target values using Rainbow beads, standardizing sample preparation and acquisition, which tend to reduce the variability of results between equipment, as already demonstrated (8-9). The longitudinal evaluation of the quality performance of the laboratories was done to verify the stability of the processes during the study, which is very important for future clinical trials, which are usually carried out over a period of years (10).

**Throughout the study, we observed that many laboratories adjusted their processes which improved their results. The persistence of intra-laboratory deviations resulting in differences between the laboratory results and the reference values suggested systematic problems related to some laboratories. The following items were identified as most frequently responsible for measurement differences: inadequate titration of monoclonal antibodies; lack of daily fluorescence adjustment using Rainbow Beads target values; the use of target values ​​from another peak instead of the 7th peak of the Rainbow beads, as recommended by the Euroflow SOPs; inappropriate cytometer compensation; lack of monitoring the stability of flow cytometer parameters through Levey-Jennings plots; variations in the MFI of the markers, from lot to lot or vial to vial, which performances must be checked before being placed in the laboratory routine; the reduction of CD45 MFI in samples processed after 24h of storage, as was recently demonstrated (11); lack of FSC and SSC adjustments that could impact the analysis; and finally difficulties in analyzing non-standard samples with marker spillovers and variations in the distribution of populations in the dot plots.**

**These troubles were reported to the laboratories during the study, and after corrective actions the laboratories were requested to submit other files for centralized analysis (Figure S2).**

Robust standardization is essential to allow the comparability of intra- and inter-laboratory data, as we have shown by the strategy used here. Well-trained professionals are essential to achieve this goal. Therefore, rigor is required to maintain the standardization of processes and allow data comparability.

**Inter-sample comparability**

**In this study, normalized MFI was not used to evaluate individual marker expressions. The small expected variations between samples prepared and acquired using the same SOPs should not impact the results, as the comparability of each parameter was made with the reference matrices of 45 samples (normal and regenerative). The used range of median and 2SD already includes inter - sample variations.**

**REFERENCES**

1. Kalina T, J Flores-Montero J, van der Velden VJH, Martin-Ayuso M, Böttcher S, Ritgen M et al. EuroFlow standardization of flow cytometer instrument settings and immunophenotyping protocols. Leukemia 2012; 26, 1986–2010

2. Theunissen P, Mejstrikova E, SedekL, van der Sluijs-Gelling AJ, Gaipa G, Bartels M et al. Standardized flow cytometry for highly sensitive MRD measurements in B-cell acute lymphoblastic leukemia. Blood. 2017;129(3):347-357

3.EuroFlow SOP for Instrument Setup and Compensation for BD FACSLyric Instruments Version 1.8. July 2019. Avaliable from [www.euroflow.org](http://www.euroflow.org)

4. Delgado JA, Guillén-Grima F , Moreno C , Panizo C , Pérez-Robles C, Mata JJ et al. A simple flow-cytometry method to evaluate peripheral blood contamination of bone marrow aspirates. Journal of Immunological Methods,  2016, 442:54-58 DOI: [10.1016/j.jim.2016.12.006](https://doi.org/10.1016/j.jim.2016.12.006)

# 5. Wood B, Jevremovic D,Bene MC, Yan M, Jacobs P, Litwin V, et al. Validation of Cell-based Fluorescence Assays: Practice Guidelines from the ICSH and ICCS – Part V – Assay Performance Criteria. Cytometry Part B 2013,84B:315–323 DOI: 10.1002/cyto.b.21108

6. Glier H, Novakova M, Marvelde J, Bijkerke A, Morf D, Thurner D, et al. Comments on EuroFlow Standard Operating Procedures for Instrument Setup and Compensation for BD FACS Canto II, Navios and BD FACS Lyric Instruments. J Immunol Methods 2019 <https://doi.org/10.1016/j.jim.2019.112680>

7. EuroFlow SOP for bulk lysis in MRD panels Version 1.3 - 25 June 2018. Available from: [www.euroflow.org/protocols](http://www.euroflow.org/protocols)

8.Keeney M, Wood BL, Hedley DB, DiGiuseppe JA, Stetler-Stevenson M, Paietta E, et al. A QA Program for MRD Testing Demonstrates That Systematic Education Can Reduce Discordance Among Experienced Interpreters. Cytometry B 2018; 94(2): 239–249. doi:10.1002/cyto.b.21528.

9.Maurer-Granofszky M , Schumich A , Buldini B , Gaipa G , Kappelmayer J , Mejstrikova E et al. An Extensive Quality Control and Quality Assurance (QC/QA) Program Significantly Improves Inter-Laboratory Concordance Rates of Flow-Cytometric Minimal Residual Disease Assessment in Acute Lymphoblastic Leukemia: An I-BFM-FLOW-Network Report. Cancers 2021, 13, 6148. doi: [10.3390/cancers13236148](https://doi.org/10.3390%2Fcancers13236148)

10. Chitteti BR, Litwin V. Instrument Installation, Operational, and Performance Qualification for BD FACS Canto II, ICCS Quality and Standards Committee, 01 Oct 2018, available on the ICCS home page, accessed online March 27, 2022

# 11. Sedek L , Flores-Montero J, van der Sluijs A, Kulis J , Marvelde J , Philippé J et al. Impact of Pre-Analytical and Analytical Variables Associated with Sample Preparation on Flow Cytometric Stainings Obtained with EuroFlow Panels. Cancers 2022, 14, 473.

**SUPPLEMENTAL TABLES**

**TABLE S1**: monoclonal antibodies (MoAb), fluorochromes, clones and manufacturers used in the study for the detection of Minimal Residual Disease in samples from patients with B-cell precursor Acute Lymphoblastic Leukemia

| **MoAb** | **Fluorochrome** | **Clone** | **Manufacturer** |
| --- | --- | --- | --- |
| CD10 | APC | HI10A | BD Biociences |
| CD19 | PECy7 | J3-119 | Beckman Coulter |
| CD19 | PECy7 | 19-1 | Cytognos |
| CD 20 | PacB | 2H7 | Biolegend |
| CD20 | HV450 | L27 | BD Biociences |
| CD20 | PacB | B9E9 | Beckman Coulter |
| CD34 | PerCPCy5.5 | 8G12 | BD Biosciences |
| CD38 | APCA750 | LS198-4-3 | Beckman Coulter |
| CD38 | APCH7 | HB7 | BD Biociences |
| CD45 | PacO | HI30 | Invitrogen |
| CD45 | V500-C | 2D1 | BD Biociences |
| CD45 | OC515 | GA90 | Cytognos |
| CD45 | OC515 | HI30 | Immunostep |
| CD45 | KrO | J.33 | Beckman Coulter |
| CD66c | PE | KOR-SA3544 | Beckman Coulter |
| CD73 | PE | AD-2 | BD Pharmingen |
| CD81 | FITC | JS-81 | BD Biociences |
| CD123 | PE | AC145 | Miltenyi |
| CD304 | PE | 12C2 | Biolegend |

**TABLE S2:** Comparison of B cell markers (MFI, within mean±2SD), cell size and complexity (inside median values), between participating laboratories and reference values

|  | | | **Initial Phase** | | | **Sequential phase** | | |  |
| --- | --- | --- | --- | --- | --- | --- | --- | --- | --- |
| **B-cell subsets** | **Parameter** | | **Total**  **N** | | **MFI mean ±2SD**  **n (%)** | **Total**  **N** | **MFI mean ±2SD**  **n (%)** | | **P-value**** |
| **Mature B cells (MFI)** | | **CD19** | 182 | 147 (81) | | 75 | | 66 (88) | 0.162 |
|  |  | **CD20** | 182 | 139 (76) | | 74 | | 67 (90) | **0.009** |
|  |  | **CD45** | 182 | 105 (58) | | 75 | | 49 (65) | 0.291 |
| **B cell Precursor**  **(MFI)** | | **CD10** | 172 | 150 (87) | | 72 | | 63 (87) | 0.950 |
|  |  | **CD19** | 172 | 165 (96) | | 72 | | 72 (100) | 0.082 |
|  |  | **CD34** | 172 | 168 (98) | | 72 | | 71 (99) | 0.638 |
|  |  | **CD38** | 172 | 158 (92) | | 72 | | 67 (97) | 0.123 |
|  |  | **CD81** | 172 | 121 (70) | | 72 | | 55 (76) | 0.337 |
|  | |  | **(Within median)*** | | | **(Within median)*** | | |  |
| **Total Lymphocytes** | | **FSC** | 195 | 76 (39) | | 77 | | 28(36) | 0.690 |
|  |  | **SSC** | 195 | 87 (45) | | 77 | | 43(56) | 0.095 |

MFI: median fluorescence intensity; SSC: side scatter; FSC: forward scatter; * Within median

of FSC and SSC target values according to EuroFlow SOPs; ** Pearson's Chi-squared test.

**SUPPLEMENTAL FIGURES**

**Figure S1:** Analysis strategy using merge files in Infinicyt software for MRD BCP-ALL detection.

**Figure S2:** Flowchart of the MRD interlaboratory standardization project of MRD in BCP-ALL

**Figure S3:** Reference matrices of the MFI average values of monoclonal markers from 45 samples (BD FACSCanto II, gray dots) and from 10 samples (BDFACSLyric, black dots), for normal B cell subsets.

**Figure S4:** An example of a degree of agreement of CD19 expression in normal B cell precursors (BCP) from 195 laboratory files in the initial phase of the study. All samples were analyzed by two independent experts (analyst 1 – blue dots, analyst 2 – orange dots). Results in agreement with the reference matrix are within the mean and 2 standard deviations (SD)

**Figure S5:** Correlation between the 2 centralized analyzes of MFI values of B cell markers from 195 files of the initial phase (A: mature B cells and B: BCP) and from 77 files of the sequential phase (C: mature B cells and D:BCP); x axis: analyst 1, y axis: analyst 2

**Figure S6:** Results of individual MFI markers of mature B cells (MBC) and B cell precursors (BCP), and averages of FSC and SSC in the initial phase **(a)** and sequential phase **(b)** of the study, corresponding to the results from 19 laboratories and 16 laboratories, respectively. Each colored dot corresponds to an individual laboratory. The 1st column corresponds to the result of analyst 1, the 2nd column to analyst 2 and the 3rd column to the reference values. Note the CD20, CD45, and CD81 MFI values outside the reference ranges, as well as the FSC and SSC scatter averages.

**Figure S7:** Bar graphs representing each laboratory in the initial and sequential phase of the study, with the percentages of concordant (green) and discordant (pink) MFI values for each marker in the normal B cell subsets (A: mature B cells, B: B cell precursors), and FSC and SSC light scatter (C)
