# Supplementary material for: Multicentric standardization of minimal/measurable residual disease in B‐cell precursor acute lymphoblastic leukaemia using next‐generation flow cytometry in a low/middle‐level income country
Source: Br J Haematol. 2022 Oct 12;200(3):381–4. doi: 10.1111/bjh.18499 (PMC10091773; doi:10.1111/bjh.18499)
Supplement: Supplementary file 9 — Table S1 [file BJH-200-381-s010.docx]

Table S1: monoclonal antibodies (MoAb), fluorochromes, clones and manufacturers used in the study for the detection of Minimal Residual Disease in samples from patients with B-cell precursor Acute Lymphoblastic Leukemia

| **MoAb** | **Fluorochrome** | **Clone** | **Manufacturer** |
| --- | --- | --- | --- |
| CD10 | APC | HI10A | BD Biociences |
| CD19 | PECy7 | J3-119 | Beckman Coulter |
| CD19 | PECy7 | 19-1 | Cytognos |
| CD 20 | PacB | 2H7 | Biolegend |
| CD20 | HV450 | L27 | BD Biociences |
| CD20 | PacB | B9E9 | Beckman Coulter |
| CD34 | PerCPCy5.5 | 8G12 | BD Biosciences |
| CD38 | APCA750 | LS198-4-3 | Beckman Coulter |
| CD38 | APCH7 | HB7 | BD Biociences |
| CD45 | PacO | HI30 | Invitrogen |
| CD45 | V500-C | 2D1 | BD Biociences |
| CD45 | OC515 | GA90 | Cytognos |
| CD45 | OC515 | HI30 | Immunostep |
| CD45 | KrO | J.33 | Beckman Coulter |
| CD66c | PE | KOR-SA3544 | Beckman Coulter |
| CD73 | PE | AD-2 | BD Pharmingen |
| CD81 | FITC | JS-81 | BD Biociences |
| CD123 | PE | AC145 | Miltenyi |
| CD304 | PE | 12C2 | Biolegend |
