# Supplementary material for: Multicentric standardization of minimal/measurable residual disease in B‐cell precursor acute lymphoblastic leukaemia using next‐generation flow cytometry in a low/middle‐level income country
Source: Br J Haematol. 2022 Oct 12;200(3):381–4. doi: 10.1111/bjh.18499 (PMC10091773; doi:10.1111/bjh.18499)
Supplement: Supplementary file 10 — Table S2 [file BJH-200-381-s001.docx]

**Table S2:** Comparison of B cell markers (MFI, within mean±2SD), cell size and complexity (inside median values), between participating laboratories and reference values

|  | | | **Initial Phase** | | | **Sequential phase** | | |  |
| --- | --- | --- | --- | --- | --- | --- | --- | --- | --- |
| **B-cell subsets** | **Parameter** | | **Total**  **N** | | **MFI mean ±2SD**  **n (%)** | **Total**  **N** | **MFI mean ±2SD**  **n (%)** | | **P-value**** |
| **Mature B cells (MFI)** | | **CD19** | 182 | 147 (81) | | 75 | | 66 (88) | 0.162 |
|  |  | **CD20** | 182 | 139 (76) | | 74 | | 67 (90) | **0.009** |
|  |  | **CD45** | 182 | 105 (58) | | 75 | | 49 (65) | 0.291 |
| **B cell Precursor**  **(MFI)** | | **CD10** | 172 | 150 (87) | | 72 | | 63 (87) | 0.950 |
|  |  | **CD19** | 172 | 165 (96) | | 72 | | 72 (100) | 0.082 |
|  |  | **CD34** | 172 | 168 (98) | | 72 | | 71 (99) | 0.638 |
|  |  | **CD38** | 172 | 158 (92) | | 72 | | 67 (97) | 0.123 |
|  |  | **CD81** | 172 | 121 (70) | | 72 | | 55 (76) | 0.337 |
|  | |  | **(Within median)*** | | | **(Within median)*** | | |  |
| **Total Lymphocytes** | | **FSC** | 195 | 76 (39) | | 77 | | 28(36) | 0.690 |
|  |  | **SSC** | 195 | 87 (45) | | 77 | | 43(56) | 0.095 |

MFI: median fluorescence intensity; SSC: side scatter; FSC: forward scatter; * Within median

of FSC and SSC target values according to Euroflow SOPs; ** Pearson's Chi-squared test.
